# Supplementary material for: Identification of photoperiod-induced specific miRNAs in the adrenal glands of Sunite sheep (Ovis aries)
Source: Front Vet Sci. 2022 Jul 22;9:888207. doi: 10.3389/fvets.2022.888207 (PMC9354845; doi:10.3389/fvets.2022.888207)
Supplement: Supplementary file 1 [file Data_Sheet_1.ZIP › Supplementary file/miRDeep2 file.zip!/miRDeep2 file/Novel_Structure/SP42A3/Novel_405.pdf]

Provisional ID : NC_019484.2_31632
Score total : 1.4
Score for star read(s) : -1.3
Score for read counts : 0
Score for mfe : 1.1
Score for randfold : 1.6
Score for cons. seed :
Total read count : 31
Mature read count : 31
Loop read count : 0
Star read count : 0

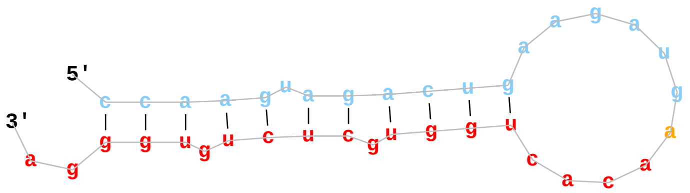


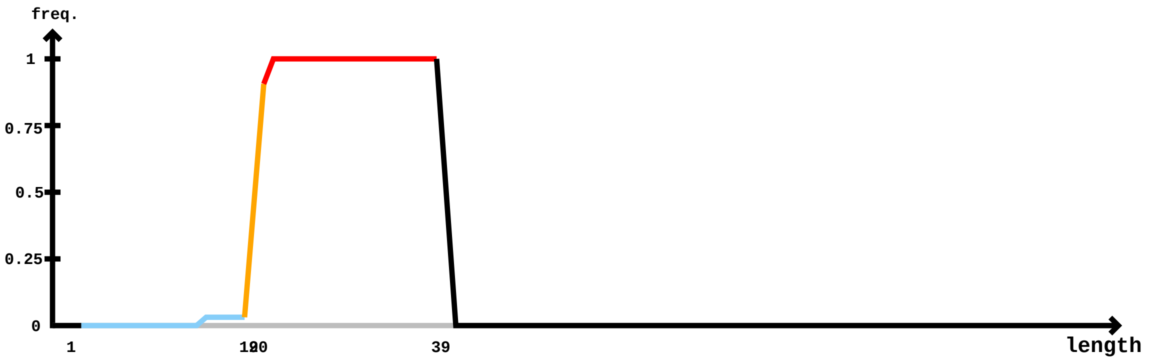


Star Mature

```
5'- cccaaguagacugaagaugaacacuggugcucuguggga ugagguaguagauuguauaguuuuagggucauaccccaucuuggagauaacuauacagucuacugucuuu   -3'    exp
    ((((((.((((((...........)))).)))).))))..((((((((((((((((((((((((((((........))))))))....)))))))))))))))))))).          reads      mm         sample
    ..............agaugaacacuggugcucuguggga......................................................................          1          0          seq
    ...................aacacuggugcucuguggga......................................................................          13         0          seq
    ....................acacuggugcucuguggga......................................................................          15         0          seq
    .....................cacuggugcucuguggga......................................................................          3          0          seq
```
